# Association of Cytokeratin and Vimentin Protein in the Genesis of Transitional Cell Carcinoma of Urinary Bladder Patients

**DOI:** 10.1155/2015/204759

**Published:** 2015-11-12

**Authors:** Arshad H. Rahmani, Ali Y. Babiker, Wanian M. AlWanian, Shawgi A. Elsiddig, Hassan E. Faragalla, Salah M. Aly

**Affiliations:** ^1^Department of Medical Laboratories, College of Applied Medical Sciences, Qassim University, Qassim 51452, Saudi Arabia; ^2^Department of Histopathology and Cytology, College of Medical Laboratories Science, University of Sciences and Technology, P.O. Box 407, Omdurman, Sudan; ^3^Faculty of Applied Medical Sciences, Aljouf University, P.O. Box 2014, Sakaka, Saudi Arabia; ^4^Faculty of Medical Laboratory Sciences, Omdurman Islamic University, 14415 Omdurman, Sudan; ^5^College of Medical Laboratories Science, Sudan University of Sciences and Technology, Khartoum, Sudan; ^6^Department of Public Health, Health Sciences College, Umm Al-Qura University, Al Leith, Makkah 21421, Saudi Arabia; ^7^Faculty of Veterinary Medicine, Suez Canal University, Ismailia, Egypt

## Abstract

The aim of study was to examine the localization and distribution of cytokeratin (CK) and vimentin protein and their association with clinical outcome of the TCC patients. Expression pattern of cytokeratin and vimentin was evaluated by immunohistochemistry in TCC cases and inflammatory lesions. Cytoplasmic expression of CK was noticed in 52.17% of TCC cases and its expression was not observed in inflammatory lesions of bladder specimens. Vimentin showed expression in 69.00% cases of TCC. Significant differences were noticed in expression pattern of CK and vimentin in inflammatory lesion and Transitional Cell Carcinoma cases. Vimentin expression increased with the grade of TCC and this difference was statistically significant whereas expression of CK decreased according to the grade of TCC. Furthermore, it was also observed that expression pattern of vimentin was high in ≥55 years as compared to <55 age group patients and these differences were significant in men as compared to women. Expression pattern of CK did not show any significant relation with age and gender. Therefore, it can be concluded that cytokeratin and vimentin will be helpful markers in the early diagnosis of Transitional Cell Carcinoma/bladder carcinoma.

## 1. Introduction

Bladder carcinoma is one of the most common malignancies worldwide in term of morbidity and mortality. Despite of its high prevalence, the molecular mechanism involved in the induction of bladder carcinoma and its progression is not properly understood [1]. Altered expressions of various genes/protein such as tumour suppressor gene, oncogene, and apoptotic genes have been observed in several types of tumour [1, 2]. A range of tumour markers and therapy targets are in use to investigate the Transitional Cell Carcinoma (TCC) and its clinical outcome. But still potential marker is needed to diagnose/investigate the Transitional Cell Carcinoma behaviour. Therefore, the assessment of potential biomarkers will be important move towards diagnosis and treatment of Transitional Cell Carcinoma. However, in this vista, intermediate filament family proteins play an important role in the genesis of Transitional Cell Carcinoma.

Cytokeratins are one of the chief structural proteins, which form the cytoplasmic network of intermediate filaments [3] and its family contains at least 20 types of cytoplasmic intermediate filaments found in epithelial cells [4]. They are expressed in a tissue-specific manner in normal organs as well as in the tumors that derived from them [5]. Different types of expression patterns of cytokeratins were noticed in carcinoma and normal/inflammatory lesions of bladder. A study report confirmed that 92% of benign/reactive cases were either CK20 (−) or (+) only in the upper 1/3 urothelium whereas in dysplastic cases CK20 staining distribution was noticed as 60% in 2/3 of the urothelium, 30% full thickness, 10% in the upper 1/3 urothelium and among carcinoma in situ (CIS) cases, 89% had full thickness of CK20 positivity [6]. Another valuable study reported that all cases (100%) of normal urothelia had normal expression patterns with Cytokeratin 20 and ninety-six percent of morphologically unequivocal cases of reactive urothelial atypia (RUA) showed normal expression patterns of Cytokeratin 20 whereas, in the carcinoma in situ (CIS) group, 86% had abnormal CK20 expression [7]. Previous finding confirmed that CK20 showed patchy cytoplasmic immunoreactivity in the superficial umbrella cell layer of the normal urothelium [8] and nonneoplastic urothelium showed no reactivity to CK20 except for umbrella cells [9].

Intermediate filaments are one of the three major cytoskeleton networks and these filaments consist of a number of different members such as vimentin and the cytokeratin proteins [10]. Vimentin shows important roles in cell adhesion, migration, and signalling [11]. Numerous studies described the vimentin reactive cells in benign and malignant breast tissues [12, 13] and vimentin expression in the tumour stroma was valuable in identifying colorectal cancer patients with a poor prognosis [14]. Different expression pattern of vimentin was also noticed in bladder cancer and normal urothelia. An important study reported that expression of vimentin was observed in 43% of bladder cancers, whereas it was not expressed or found negative in all normal urothelia [15]. The aim of study was to assess the expression profile of cytokeratin and vimentin in Transitional Cell Carcinoma and its association with clinical outcome such as sex, age, and grade of the tumour via immunohistochemistry.

## 2. Materials and Methods

### 2.1. Tissue Specimens

Forty-six patients with Transitional Cell Carcinoma (TCC) and ten cases of inflammatory lesions of bladder, confirmed by histopathologist, were taken to examine the expression profile of both markers and its interpretation with clinical outcome. The patient's details about age and sex were noted as range of 24–78 years with mean age 36 ± 12 years and 38 male and 8 female cases. The TCC cases were further categorized as Grade I (*n* = 14), Grade II (*n* = 18), and Grade III (*n* = 14). Haematoxylin and eosin (H and E) staining was performed on each case to confirm the grading of the tumour.

### 2.2. Immunohistochemical Detection of Cytokeratin and Vimentin Protein

Transitional Cell Carcinoma (TCC) was sectioned with microtome into 5 *μ*m thick. Deparaffinized was performed with three changes of xylene and blocking of endogenous peroxidase activity was done via 0.3% hydrogen peroxide in methanol for 30 minutes. After the quenching step, antigen retrieval was made with citrate buffer (pH 6.0) in pressure cooker for 30 minutes and then sections were kept at room temperature for 20–30 minutes. Then, blocking step was made with blocking agent for 10 minutes and sections were washed with PBS. Monoclonal mouse anti-human cytokeratin (Clone AE1/AE3, Dako) and Monoclonal mouse Anti-Vimentin (Clone V9, Dako) antibodies was applied at 1 : 100 and 1 : 75 dilutions for 2 hours at room temperature in humid chamber. Following incubation with secondary antibody for 45 minutes, followed by incubation with streptavidin-biotin enzyme complex was applied for 30 minutes. Finally, diaminobenzidine (DAB) chromogen was used and then sections were counterstained with haematoxylin.

### 2.3. Evaluation of Staining

Negative (omission of antibody) and positive controls (oral cancer) cases were run to verify the quality of staining and confirmation of the procedures. Markers such as cytokeratin and vimentin were considered as positive if more than 5% of cells were positive and less than 5% cytoplasmic positivity was taken as negative. Cytoplasmic expression of cytokeratin and vimentin was considered as positive cases. All fields of the section were analyzed by two pathologists and more than 400 tumor cells, in five different area, were counted and mean percentage was calculated.

### 2.4. Statistical Analysis

Markers' expression and its association with clinical outcome were analyzed by Chi square (*λ*)^2^. The *P* value *P* < 0.05 was considered as statistically significant.

## 3. Results

Cytokeratin and vimentin markers were analyzed according to age, sex, and histological grade and results were interpreted accordingly.

### 3.1. Immunohistochemical Analysis of Cytokeratin Protein

**Figure 1:**
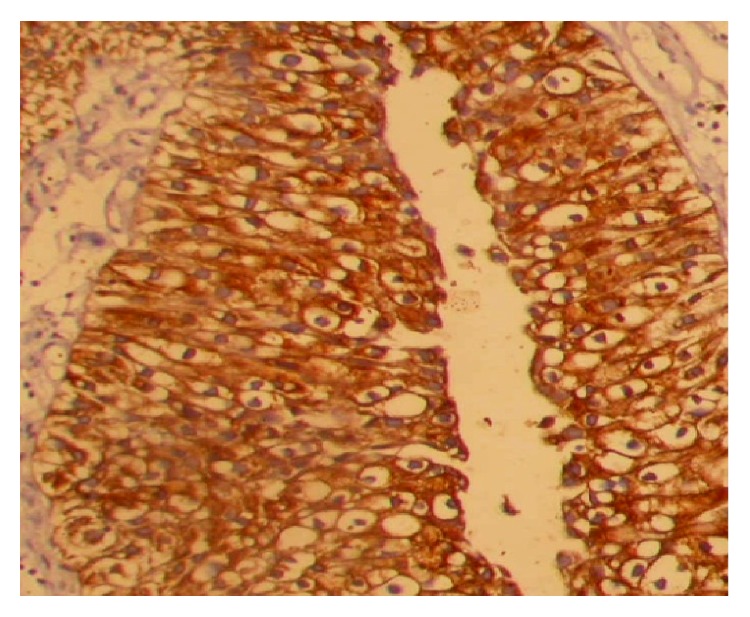
Cytoplasmic expression of cytokeratin protein in Transitional Cell Carcinoma (orig. mag ×100).

**Figure 2:**
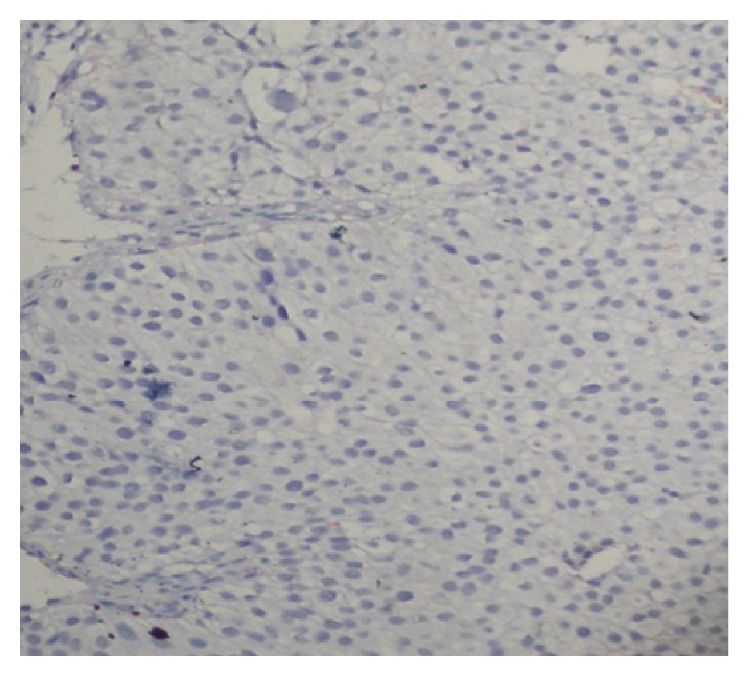
Cytokeratin did not show expression in Transitional Cell Carcinoma (orig. mag ×100).

Cytokeratin expression was noticed in 24.00 (52.17%) of TCC cases in cytoplasm (Figure 1) including 9 (64.28%) in Grade I, 10 (55.55%) in Grade II, and 5 (35.71%) in Grade III whereas 22.00 (47.82%) of TCC did not show any expressions of cytokeratin protein (Figure 2). Furthermore, the expression profile was examined according to age and sex and difference of expression pattern was insignificant (Table 1). Markedly, the positivity of CK decreased according to grade of the tumour, but this difference was statistically significant. Among inflammatory lesions of bladder cases, CK did not exhibit any expression. The expression pattern of CK was measured in both sexes and less than 50 years and equal to or more than 50 years age group, but differences in expression pattern of CK did not reach statistically significant level in gender and different age groups.

### 3.2. Positivity of Vimentin in TCC and Inflammatory Lesions of Bladder Cases

**Figure 3:**
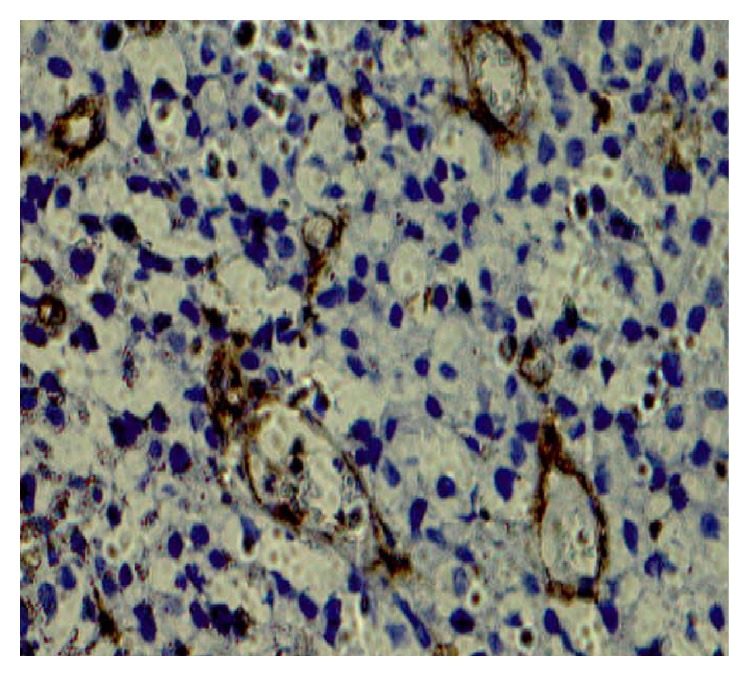
Vimentin showed expression in Transitional Cell Carcinoma (orig. mag ×100).

**Figure 4:**
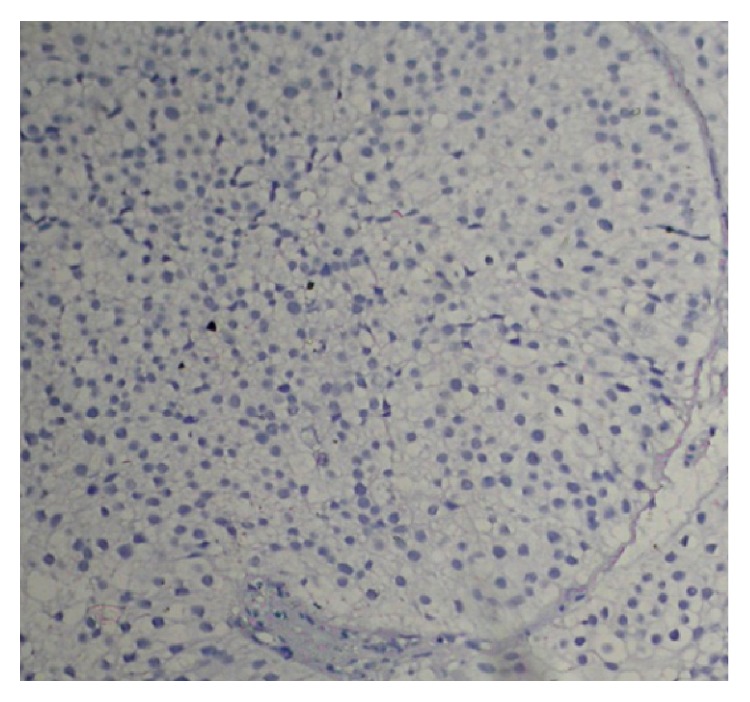
Vimentin did not show expression in Transitional Cell Carcinoma (orig. mag ×100).

Vimentin expressions were analyzed in TCC cases and it was noticed that vimentin was overexpressed in 32 (69.56%) of TCC cases (Table 1 and Figure 3) and 14 cases (30.43%) did not show any expression of vimentin (Figure 4). One case out of 10 showed expression in inflammatory lesions of bladder. This difference of expression pattern in TCC and inflammatory lesions was statistically significant. Expression of vimentin was further categorized according to grade, gender, and sex. The positivity of vimentin increased according to grade of the TCC and it was (6 cases, 42.85%), (13 cases, 72.22%), and (13 cases, 92.85%) in Grade I, Grade II and Grade III, respectively (Table 1). The expression pattern of vimentin was statistically significant according to grade. The TCC cases were divided into two groups on the basis of age, that is, <55 years and ≥55 years, and it was observed that expression of vimentin was high in ≥55 years as compared to <55 years and these differences were significant in men as compared to women (Table 1).

### 3.3. Correlation of Both Markers and Their Interpretation according to Age and Gender

Transitional Cell Carcinoma (TCC) cases were examined for both markers; most of the cases showed both CK and vimentin positivity and our results showed that cytokeratin and vimentin have pivotal role in development and progression of TCC. A negative correlation was observed in CK and vimentin expression with gradewise observations. Markedly, the positivity of CK decreased according to grade of TCC whereas vimentin increases according to the grade of the carcinoma.

## 4. Discussion

Bladder cancer is the fifth most common cancer worldwide and also one of the major causes of cancer morbidity and mortality [16]. It affects men more as compared to women (3 : 4.1) [17]. Our finding also showed similar pattern and most of TCC patients were male (38 cases, 82.60%) and (8 cases, 17.39%) were female. The exact reason behind this is not understood, but it is thought that males acquire smoking, chewing, and drugs habits earlier than women. Currently, various markers are in practice to diagnose the Transitional Cell Carcinoma via immunohistochemistry. But still a potential marker is needed to diagnosis the early bladder carcinoma/Transitional Cell Carcinoma. In this vista, our study tried to find some more information of CK and vimentin role in the diagnosis of bladder carcinoma/Transitional Cell Carcinoma.

In our finding expression of cytokeratin was noticed in 24 cases of TCC (52.17%) and CK was not expressed in inflammatory lesion of bladder cases. Furthermore, our results showed that the expression pattern of CK decreased according to the grade of the TCC (from Grades I to III) and these differences were statistically significant. Several findings showed different types of results in this issue and some results were in accordance with our study. One of the study results revealed that positivity of Cytokeratin 20 associated with increasing tumor grade and stage and it was observed that 69.4% cases of high grade tumors showed Cytokeratin 20 positivity as compared to 45.00% of other grades of tumours [18]. Another study reported that CK-20 expression was observed in all grades of tumour as 75.00% of low malignancy potential, 83.00% of low grade, 38.00% of high grade, and 67.00% of high grade tumors that invaded adjacent structures [19]. Other important study results showed that reduction or loss of cytokeratin expression was significantly correlated with tumor stage and grade [20]. Our finding did not reach significant level in terms of positivity of CK in gender and age basis analysis.

In the current study vimentin expressions were only noticed in cytoplasm and 32 cases (69.56%) of TCC showed expression of vimentin. Previous finding also showed high expression in Transitional Cell Carcinoma cases. A study finding showed that 30 (24.8%) were positive for vimentin in bladder cancer patients [21] and other important investigations showed that 43.00% bladder cancer expressed vimentin [15].

The present study results confirmed that vimentin expression is associated with the grade of the tumour. A recent study showed that expression of HMGA2, loss of E-cadherin, and expression of vimentin are significantly correlated with bladder cancer grade and stage [15]. Another study also confirms that vimentin was found to have statistically significant correlations with grade, recurrence, and progression [21]. Our results show that expression was different in different age group and it was noticed that older age group (≥55) showed high expression (63.00%) of vimentin especially in male. The exact reason is difficulty to explain the difference in expression pattern of both markers in cancer and inflammatory lesions. But it is thought that long cumulative exposure of carcinogen plays a critical role in the DNA damage/DNA adduct formation and alterations in transitional epithelial. The current study demonstrates that significant difference of expression pattern of both markers in TCC and inflammatory lesions of bladder and expression of vimentin was closely associated with the grade of TCC. Keeping in view the above information, our study concluded that use of both markers will be helpful in the diagnosis/investigation of early Transitional Cell Carcinoma.

## Figures and Tables

**Table 1:** Expression of cytokeratin (CK) and vimentin in TCC and inflammatory lesion cases and its association with grade of the tumour.

Clinical parameters patients	CK expression				Vimentin expression	
	Total cases	% positivity	*P* value	Positive cases	% positivity	*P* value
Inflammatory lesions	10	00		01	10.00	
Tumor grades						
I	9	64.28	<0.05	6	42.85	<0.05
II	10	55.55		13	72.22	
III	5	35.71		13	92.85	
Total cases	24	52.17		32	69.56	
Sex						
Male	20	52.60	>0.05	31	81.00	<0.05
Female	04	50.00		01	12.5	
Age						
<55 years	8	50.00	>0.05	7	43.00
≥55 years	16	55.00		25	63.3
